# Influence of Renal Function and Age on the Pharmacokinetics of Levofloxacin in Patients with Bone and Joint Infections

**DOI:** 10.3390/antibiotics9070401

**Published:** 2020-07-10

**Authors:** Gauthier Eloy, David Lebeaux, Manon Launay, Marie-Paule Fernandez-Gerlinger, Eliane Billaud, Emmanuel Douez, Jean-Luc Mainardi, Benjamin Bouyer, Vincent Jullien

**Affiliations:** 1 Service d’Orthopédie et de Traumatologie, Hôpital Européen Georges Pompidou, Assistance-Publique-Hôpitaux de Paris, 75015 Paris, France; gauthier.eloy@aphp.fr (G.E.); benjamin.bouyer@aphp.fr (B.B.); 2 Unité Mobile de Microbiologie Clinique, Service de Microbiologie, Hôpital Européen Georges Pompidou, AP-HP, 75015 Paris, France; david.lebeaux@aphp.fr (D.L.); marie-paule.gerlinger@aphp.fr (M.-P.F.-G.); jean-luc.mainardi@aphp.fr (J.-L.M.); 3 Faculté de Médecine, Université Paris Descartes, 75015 Paris, France; Manon.Launay@chu-st-etienne.fr (M.L.); eliane.billaud@aphp.fr (E.B.); 4 Hôpital Européen Georges Pompidou, Service de Pharmacologie, Assistance-Publique-Hôpitaux de Paris, 75015 Paris, France; edouez01@gmail.com; 5 Unité Fonctionnelle de Pharmacologie, Groupe Hospitalier Paris Seine Saint-Denis, UFR SMBH, Université Paris 13, 93000 Bobigny, France

**Keywords:** levofloxacin, bone and joint infection, pharmacokinetics, glomerular filtration rate

## Abstract

Despite its efficacy and toxicity being exposure-related, levofloxacin pharmacokinetics in patients with bone and joint infections has been poorly described to date, so the possible need for a dose adjustment is unknown in this population. A prospective population pharmacokinetic study was conducted in 59 patients to answer this question. The final model consisted of a one-compartment model with first-order absorption and elimination. Mean parameter estimates (% interindividual variability) were 0.895 h^−1^ for the absorption rate constant (Ka), 6.10 L/h (40%) for the apparent clearance (CL/F), 90.6 L (25%) for the apparent distribution volume (V/F). Age and glomerular filtration rate (GFR), estimated by the modification of diet in renal disease formula, were related to CL/F by power models, and CL/F was found to increase for increasing GFR and decreasing age. For a similar GFR, the simulated area under the curve (AUC) was 55% higher in 70 years-old patients compared to 30 year-old patients. Based on this model, a 750 mg dose should provide an optimal exposure (AUC/ minimum inhibitory concentration (MIC) ≥100), with the possible exception of patients older than 60 years and with GFR <70 mL/min/m² who may necessitate a dose reduction, and patients with infections caused by bacteria with MIC close to 1 mg/L who may need an increase in the dose.

## 1. Introduction

Levofloxacin is a broad spectrum antibiotic belonging to the fluoroquinolone class, and corresponding to the active enantiomer of ofloxacin [1]. Levofloxacin is well absorbed, with an absolute bioavailability of around 99%, extensively diffuses into many tissues and body fluids, and is 24–38% bound to plasma proteins, mainly albumin. Levofloxacin displays linear pharmacokinetics and is 80% excreted unchanged via the kidneys. However, renal clearance is 60% higher than creatinine clearance, evidencing the involvement of tubular secretion [2]. Levofloxacin also undergoes an hepatic metabolism, but the metabolites are not thought to contribute to the antimicrobial efficacy of the parent drug [3,4].

Levofloxacin exhibits good penetration into bone, with mean bone/plasma ratios of 1 and 0.5 for cancellous and cortical bone, respectively [5]. Consequently, levofloxacin is a good candidate for the treatment of bone and joints infections, more particularly since doses of 500 and 750 mg per day provided good outcomes [6,7]. It is, however, well known that fluoroquinolones efficacy is related to the area under the curve/minimum inhibitory concentration against the causative bacteria (AUC/MIC) ratio, with a currently accepted target of around 100–125 [8,9,10,11]. For example, a clinical pharmacokinetic/pharmacodynamic (PK/PD) study performed in 134 hospitalized patients with proven skin, respiratory, or complicated urinary tract infection evidenced that patients with AUC/MIC ratio >100 (or peak/MIC > 12) had only a 1% risk of clinical failure, compared to risks of 12 and 43% for patients with AUC/MIC ratios of 25–100 and <25, respectively [12,13]. A similar AUC/MIC cut-off of 96 was also recently identified in hospitalized elderly patients with acute infections [14]. It is therefore important to determine the factors explaining the interindividual pharmacokinetic variability of levofloxacin in order to provide an optimal exposure to this drug and reduce the risk of treatment failure. 

Several population pharmacokinetic models have been published to date in healthy subjects and in patients [14,15,16,17,18,19,20,21,22,23,24,25,26]. In these models, the covariates found to be related to levofloxacin clearance were creatinine clearance (CLcr) [14,15,16,17,18,19,26], body weight (BW) [15], age [15,16,20], and race [16]. However, none of these studies were conducted in patients with bone and joint infections, so it is uncertain whether these models could be applied to the determination of appropriate dosing regimens in these patients.

We therefore decided to conduct a population PK study of levofloxacin in patients with bone and joint infections in order to verify whether the current dose recommendations provide a satisfying exposure or if it could be improved in some patients according to the relevant covariates.

## 2. Results

### 2.1. Patients and Collected Data

Fifty-nine patients (28 men) provided the data. Their physiological parameters are displayed in Table 1. 

The value of weight was unavailable for 1 patient, CLcr for 1 patient, C-reactive protein for 2 patients, proteinemia for 6 patients, bilirubin for 14 patients, hepatic enzymes for 15 patients, and albumin for 51 patients. Consequently, albumin was not investigated as a covariate. Thirty-one patients received concomitant rifampin. One hundred and ninety-seven samples were available for PK evaluation. Different dosing regimens were observed at the sampling time: 5 patients received 500 mg once a day, 45 patients received 750 mg once a day, 8 patients received 500 mg twice a day, and 1 patient received 750 mg twice a day. The most frequent clinical context was spine surgical infection, which was observed in 33 patients. Forty-four patients had surgical materials. The period between surgery and the first levofloxacin sample was twelve days. 

### 2.2. Pharmacokinetic Modeling

The best structural model was a one-compartment model with first-order absorption and elimination. Interindividual variability (IIV) could be estimated for the apparent clearance (CL/F) and distribution volume (V/F), but not for the absorption rate constant (Ka), so no covariate could be investigated for this parameter. Additionally, a covariance between the etas of V/F and CL/F significantly decreased the objective function. This covariance was nevertheless fixed to its estimated value with the base model, since its relative standard error was high. The residual error model was proportional and no interoccasion variability could be estimated. Eta shrinkage for CL/F and V/F was 9 and 41%, respectively. Epsilon shrinkage was 14%. Concerning covariate analysis, age, glomerular filtration rate (GFR), and C-reactive protein significantly decreased the objective function and were found to explain 45, 35, and 14%, respectively of the IIV of CL/F during the forward selection. However, only age and GFR could be maintained in the model after the backward process. No covariate was found to explain the IIV of V/F. The final model was therefore:

Ka (h^−1^) = 0.895

CL/F (L/h) = 6.1 × (AGE in years/58)^−0.52^ × (Glomerular Filtration Rate in mL/min/1.73m²/105)^0.48^

V/F (L) = 90.6

Interestingly, when CLcr was used instead of GFR, age could not be maintained in the model. The obtained equation was: CL/F = 6.76 × (CLcr/120)^0.73^. However, the model with GFR and age was considered as the final model because (i) GFR was known for all patients, (ii) it provided a slightly lower objective function (494.1 vs 495.9), (iii) the MDRD formula is known to be less biased and more precise than the Cockcroft–Gault equation [27] and (iv) it better predicted the mean clearances observed in previously published studies, more particularly in young subjects (see Supplemental Table S1). 

All estimation and bootstrap results for the final model are provided in Table 2. 

**Figure 1:**
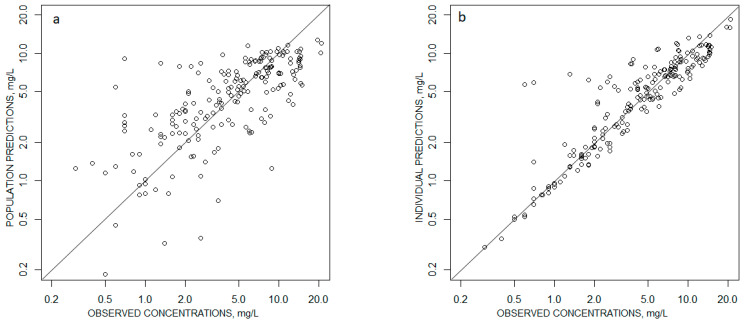
Goodness of fit curves representing (**a**) the Population Predictions vs observed concentrations and (**b**) the individual predictions vs observations. Solid diagonal line: x = y line.

**Figure 2:**
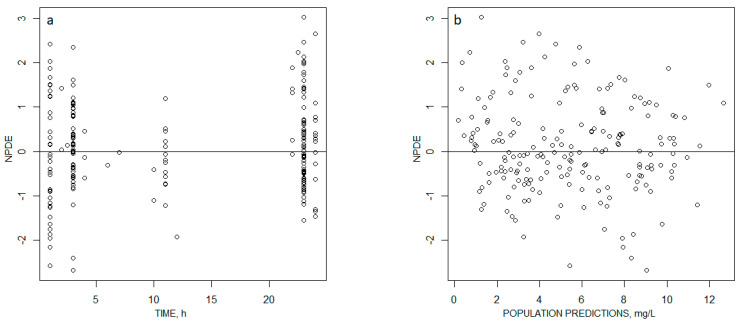
Normalized prediction errors (NPDE) with respect to (**a**) time after dose, and (**b**) population predictions.

**Figure 3:**
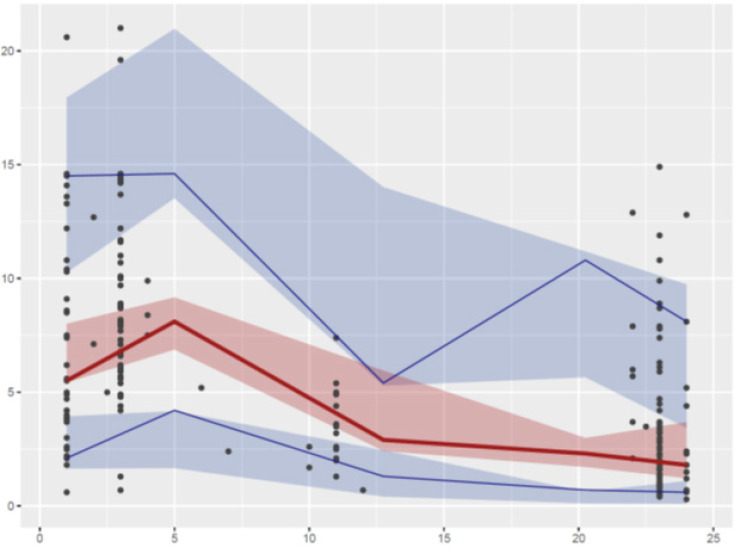
Visual predictive checks with the final model. Circles: observed concentrations, upper blue, median solid red, and lower blue lines: 97.5th, 50th, and 2.5th percentiles obtained from the observed concentrations, respectively. Lower blue, median red, and upper blue areas are: 95% confidence interval of the predicted 2.5th, 50th, and 95th percentiles, respectively.

Relative standard deviations >50% were obtained with the bootstrap for the IIV of V/F and the covariance between the etas of V/F and CL/F. No bias was observed on the graphs displaying the population predictions or individual predictions with respect to observed concentrations (Figure 1), the normalized prediction errors with respect to population predictions or time after dose (Figure 2), or the visual predictive checks (Figure 3). 

### 2.3. Dose Evaluation

The AUC obtained for the usual 750 mg daily dose was simulated with respect to age and GFR. Three ages and five different GFR were investigated (30, 50, and 70 years; 40, 70, 100, 130, 160 mL/min/1.73 m²). Dose modifications are recommended for CLcr < 50 mL/min; however, in order to differentiate the influence of age in patients with a GFR close to the limit value, we decided to evaluate a GFR of 40 mL/min/1.73 m² without dose adjustment. Simulated AUCs are displayed in Table 3. Of note, a three order magnitude was observed between the lowest and highest mean AUC (79 mg h/L for 30 years old patients with a GFR = 160 mL/min/1.73 m² vs 233 mg h/L for 70 year-old patients with a GFR equal to 40 mL/min/1.73 m²). Additionally, for a similar GFR, 70 year-old patients have an AUC 50–60% higher than a 30 years-old subject. For instance, it can be seen that a 70 year-old patient with a GFR = 100 mL/min/1.73 m², will likely have a similar AUC than a 30 year-old patient with a GFR = 40 mL/min/1.73 m² (i.e., around 150 mg h/L).

The probability to achieve a target AUC/MIC = 100 for a MIC equal to 1 mg/L is provided Table 4. It can be seen this probability can be low, more particularly for 30 years-old patients and older patients with a high GFR ≥ 70 mL/min/1.73 m². For a lower MIC of 0.5 mg/L, only 30 year-old patients with a GFR ≥ 100 mL/min/1.73 m² have a suboptimal PTA (Table 5). For a MIC of 0.25 mg/L, all PTAs are equal to 100%. 

## 3. Discussion

The present study investigated the pharmacokinetics of levofloxacin in patients with bone and joint infections. A one-compartment model with first-order absorption and elimination appropriately described the data. Levofloxacin PK was already described with a one-compartment model with first-order absorption and elimination [15,17,20,24,25,28,29], but other studies used two-compartment models [14,16,17,18,22,23,26,30]. It is likely that our sampling schedule was too sparse to allow the identification of more than one compartment. It is also the likely reason for the quite high uncertainty we obtained for the interindividual variability of V/F (Table 2). However, this does not seem to have penalized our clearance model. Indeed, if our mean estimate for CL/F (6.10 L/h) was lower than the value described in patients with community-acquired infections (9.27 L/h) [16], in critically ill patients (8.66 L/h) [26], and in healthy volunteers (10.8 L/h) [25], and was higher than the value observed in elderly subjects (2.53 L/h) [14], while it was similar to the values described in patients with tuberculosis (7.63 L/h) [17], patients with prostatitis (7.27 L/h) [22], Chinese patients with infections (5.8 l/h) [18], patients with febrile neutropenia (5.8 L/h) [28], Korean patients (6.19 L/h) [19], and in another study in healthy volunteers (5.97 L/h) [23]. Between-study comparisons are nevertheless challenging because of the possible differences in the administration routes and in the demographic characteristics of the included subjects. In addition, the renal function was evaluated by different formulas from one study to another. For instance, the study in healthy volunteers which found a mean CL/F = 10.8 L/h included younger subjects (mean age = 28 years and mean GFR = 114 mL/min/1.73 m²) and the study in elderly was characterized by a higher mean age of 81 years and a lower mean GFR of 30 mL/min/1.73 m². Interestingly, using these demographic values with our model led to very similar clearance values of 9.27 and 2.82 L/h for the healthy volunteers and the elderly patients, respectively. Also, our model allowed for a satisfying prediction of CL/F for five out of seven studies which used CLcr as a marker of the renal function. It also can be noted that one of the two studies for which our model performed poorly was about critically ill patients, a context known to be associated with major PK changes [31]. We believe this overall consistency between the present results and previous population PK studies, displayed in Supplemental Table S1, supports the robustness of our clearance model, allowing its use for the prediction of levofloxacin AUC. 

Rifampin, despite being a powerful enzymatic inducer, was not found to have a significant effect on the CL/F of levofloxacin. Two reasons may explain this result: (i) levofloxacin is mainly eliminated via glomerular filtration, and (ii) the blood samples data were obtained 2 or 3 days after the beginning of treatment, whereas the inductive effect of rifampin increases progressively during the first 10 days of treatment [32], so the possible induction of the mechanisms of excretion of levofloxacin other than GFR may have been underestimated. However, to our knowledge, no significant interaction was identified to date between these two drugs.

An interesting result is the simultaneous presence of age and GFR as covariates explaining the IIV of CL/F in the final model, despite these two covariates being correlated (r = 0.64 in our population). This result suggests that other mechanisms than GFR involved in the elimination of levofloxacin (tubular secretion and metabolic transformation) could also evolve with age and be more efficient in young patients. A consistent result was nevertheless previously described, as Preston et al. also found that levofloxacin CL/F increased with increasing CLcr and independently decreased with increasing age [16]. However, when we used CLcr instead of GFR, the influence of age on CL/F did not remain significant. This result confirms that the MDRD formula and the Cockcroft–Gault equation may not be strictly equivalent to determining the glomerular filtration rate [27]. This result also highlights the complexity of dose adjustment for levofloxacin. Currently, levofloxacin dose recommendations are based on the CLcr value in mL/min, and a reduction in the dose by 50% is recommended for a CLcr comprised between 20 and 49 mL/min. According to our results, these recommendations should be modified when the MDRD formula is used to evaluate the renal function, as is the case in our institution. Indeed, a 70 year-old patient with a normal GFR of 100 mL/min/1.73 m² will have an exposure to the drug equivalent to the one that would be observed in a 30 years-old subject with a suboptimal GFR of 40 mL/min/1.73 m². Using the MDRD formula combined with current recommendations, an unnecessary dose reduction would occur in the second case. Avoiding a too-high exposure is an important issue, since levofloxacin is the fluoroquinolone associated with the highest risk of tendinopathy [33]. This toxicity seems to be exposure-related, and several risk factors were identified: concomitant corticosteroid therapy, history of solid organ transplantation, renal impairment, and age >60 years [33]. Our results seem to confirm that an old age could be associated with an increase in the exposure leading to an increased risk of tendinopathy. Based on the equation we obtained with CLcr as a covariate, a mean AUC of 210 mg h/L can be expected in patients with a CLcr = 50 mL/min so, according to the simulated AUC values displayed in Table 3, a dose reduction to 500 mg could be proposed to patients older than 60 with a GFR comprised between 40 and 70 mL/min/m² and to 50 years-old patients with a GFR = 40 mL/min/1.73 m². Conversely, a systematic dose reduction seems unnecessary for patients younger than 50 years with GFR ≥ 40 mL/min/m². Dose adjustment should also take efficacy into account, keeping in mind that determining a PK/PD target in the context of bone and joint infections is challenging since the measurement of the concentration of the drug at the infection site is extremely complex [34]. In the present study, we decided to consider the bone to plasma ratio of 1 that was determined for cortical bone in a previous study [5]. Our Monte Carlo simulations evidenced that a daily dose of 750 mg would provide a low probability to achieve the PK/PD target for a MIC equal to the clinical breakpoint for *Staphylococcus aureus* (1 mg/L), so an increase in the dose could be considered (Table 4). However, only 1.2% of the *S. aureus* strains have a MIC = 1 mg/L, so a systematic increase in the dose cannot be recommended based on the above-mentioned safety concern. In addition, the high PTA observed for MIC < 1 mg/L confirmed the 750 mg daily dose provides an optimal exposure for more than 95% of the patients with *S. aureus* infections. Some studies suggested the diffusion of levofloxacin into cancellous bone could be twice lower as in cortical bone, which would raise the target AUC/MIC to 200 [5,35]. In this case, the 750 mg daily dose would provide low PTAs for MICs of 1 and 0.5 mg/L, and the PTAs for a MIC = 0.25 mg/L would be equal to the results displayed in Table 5, while PTAs = 100% would be found for MICs ≤ 0.125 mg/L. Since 93% of *S. aureus* strains have a MIC ≤ 0.25 mg/L according to EUCAST data, it can also be concluded that the daily dose of 750 mg would provide an optimal exposure in a high proportion of patients suffering from an infection of the cancellous bone due to *S. aureus*. Based on these results, only infections due to bacterial strains with high MICs of 0.5–1 mg/L could necessitate an increase in the dose. However, if one considers infections due *to Pseudomonas aeruginosa*, as it was the case for four patients in the present study, it appears this increase in the dose would be frequently necessary as 68% of *P. aeruginosa* strains have a MIC ≥ 0.5 mg/L according to EUCAST data. 

We believe that these results highlight the necessity to individualize the treatment by considering the age and GFR of the patients concomitantly with the microbiological context and the localization of the infection site. Therapeutic drug monitoring also appears to be an interesting tool, more particularly in elderly patients or in the case of infections caused by bacteria with high MICs.

## 4. Materials and Methods

### 4.1. Patients and Collected Data

The study protocol was approved by the local ethics committee (Commité d’éthique pour la recherche AP-HP.5, IRB registration # 00011928). Routine data were prospectively collected from patients hospitalized at the Hôpital Européen Georges Pompidou between April 2016 and December 2017 for the treatment of a bone and joint infection. Patients with renal impairment requiring extra renal replacement therapy were excluded. Blood samples were drawn after ≥2 or 3 days of treatment and consisted of a trough concentration followed by 3 supplemental samples: 1 hour after the following intake, 3 hours after the intake, and just before the next intake. Demographic data were recorded: age, BW, gender, combined treatments, as well as biological data, if available on the same day as the samples: proteinemia, albuminemia, serum glutamic pyruvic transaminase, serum glutamic oxaloacetatic transaminase, C-reactive protein, and glomerular filtration rate (GFR, expressed in mL/min/1.73 m²) calculated by the Modification of Diet Renal Disease formula [36]. When possible, creatinine clearance (CLcr, expressed in mL/min) was calculated using the Cockcroft–Gault equation [37]. Microbiological and clinical data were also recorded: causative bacteria, type of bone and joint infection, anatomical site, presence of surgical materials. 

### 4.2. Levofloxacin Assay

All blood samples were centrifuged within 2 hours and the plasma stored at −20 °C until analysis, which was performed twice weekly. The sample assay consisted of a validated LC-MS/MS method. Briefly, 50 µL of desionized water and 50 µL of the internal standard (i.e., deuterated zolpidem) were added to 50 µL of the plasma sample. Protein precipitation was performed by adding 75 µL of trichloroacetic acid 5%. Samples were then vortex-mixed and centrifuged, and 50 µL of the supernatant were diluted with 200 µL of ammonium acetate 0.2 M before injection into the chromatographic system. Intra- and interday biases and imprecisions were inferior to 15% over the calibration range of the method (0.1–24 mg/L). 

### 4.3. Population PK Modeling

Concentration–time data were analyzed by use of the first-order conditional estimation with the interaction method of the non-linear mixed effects modelling program NONMEM (version 7.3). Several structural pharmacokinetic models were investigated. Classical one- and two-compartment models with several error models (i.e., proportional, exponential, and additive random effects model) were investigated as means of describing interpatient and residual variabilities. Interindividual variability (IIV) was described by assuming that individual parameters arise from a multivariate lognormal distribution with mean vector and variance-covariance matrix to be estimated. Interoccasion variability was also investigated as exponential models. Systematic testing for the influence of continuous covariates on the pharmacokinetic parameters (P) was done by the use of a generalized model, according to the following equations:

(i) P = TV(P) × (COV/mean COV)^θ_COV_^,

where TV(P) is the typical value of the apparent PK parameter for a patient with the mean covariate (COV) value, and θ_COV_ is the corresponding influential factor. GFR was the main covariate used to reflect the renal function. However, CLcr was also tested.

Categorical covariates (combined drug, gender) were investigated according to the following equation:

(ii) P = TV(P) × θ_COV_,

where θ_COV_ was estimated for patients displaying the covariate (combined drug, male patients) and was otherwise fixed to 1. When a covariate was supposed to be related simultaneously to several PK parameters (for instance albumin or protein on clearance and distribution volumes), the same influential factor was estimated on all tested parameters. In case the value of a given covariate was not known in every patient, the covariate analysis was first performed on the subset of patients for those for whom the covariate value was known, and secondly on the entire population, the corresponding influential factor being set to the mean value for patients whose covariate value was unknown. At this step, the influence of the covariate still had to be significant in order to be kept in the model.

The significance of a relationship between a pharmacokinetic parameter and a covariate was assessed by the use of the chi-square test of the difference between the objective functions of the basic model (without the covariate) and the model with the covariate. During the forward selection process, a covariate was retained in the model if it produced a minimum decrease in the objective function of 4 units (*P* = 0.05, 1 degree of freedom) and if its effect was biologically plausible. An intermediate multivariate model including the covariates that significantly decreased the objective function was then obtained. The backward selection process was then performed, and a covariate was retained in the final multivariate model if its deletion from the intermediate model led to a 7-point increase in the objective function (*P* = 0.01, 1 degree of freedom). At each step, the goodness of fit was evaluated by use of a graph of the weighted residuals versus time after administration of the dose (time) or by use of a graph of the weighted residuals versus the predicted concentration. The accuracy and robustness of the final population model were assessed by a prediction and variability-corrected visual predictive check (pvcVP) and by a bootstrap based on 500 resamplings of the original dataset [38,39]. Lack of bias was also evaluated by inspection of the normalized prediction errors (NPDE) with respect to time after dose and population predictions (PRED) [40]. Goodness of fit was also visualized on the graphs displaying the PRED or individual predictions (IPRED) vs observed concentrations (OBS).

### 4.4. Dose Evaluation

The final model was first used to determine the area under the curve over 24h (AUC) that was obtained with the usual 750 mg per day dose according to the relevant covariates. For this, 4000 patients were simulated for each investigated combination of covariate level. Using the same methodology, the final model was then used to determine the probability to achieve an AUC/MIC target of 100 with the currently used 750 mg daily dose, according to the relevant covariates. This target assumed a bone to plasma concentration ratio of 1, as it was previously suggested for cancellous bone [5]. Since methicillin-susceptible *Staphylococcus aureus* is the most frequent bacteria against which levofloxacin is prescribed in the context of bone and joint infections, the corresponding EUCAST clinical breakpoint of 1 mg/l was chosen as the first investigated MIC. However, because 98.8% of *Staphylococcus aureus* have a MIC < 1 mg/L according to EUCAST data, lower MICs of 0.5 and 0.25 mg/L (5.4 and 36.6% of *Staphylococcus aureus*, respectively) were also investigated.

## 5. Conclusions

This study investigated the pharmacokinetics of levofloxacin in patients with bone and joint infections by the use of a population approach. The model evidenced that the apparent clearance decreased with decreasing glomerular filtration rate and with increasing age. The current 750 mg daily dose seems appropriate for the majority of the patients with bone and joints infections due to *S. aureus*. Nevertheless, a decrease in the dose could be evaluated for patients >60 years with GFR ≤ 70 mL/min/1.73 m², except if the infection is due to bacteria with a high MIC of 1 mg/L. No decrease in the dose seems necessary for patients <50 years of age and with a GFR ≥ 40 mL/min/m². On the other hand, an increase in the dose could be evaluated when the infection is caused by bacteria with a MIC of 0.5–1 mg/L.

## Figures and Tables

**Table 1:** Physiological parameters of the patients.

Clinical Characteristics	
Age (mean ± SD)	57.5 ± 20.1
BW, kg (mean ± SD)	72.1 ± 15.9
**Diagnosis**	
Spine surgical site infection, *n* (%)	33 (57%)
Limb surgical site infection, *n* (%)	19 (33%)
Osteoarthritis, *n* (%)	5 (9%)
Spondylodiscitis, *n* (%)	1 (1%)
**Surgical Materials**	
Yes, *n* (%)	44 (76%)
No, *n* (%)	14 (24%)
**Microbiologic Characteristics**	
Monomicrobial infection, *n* (%)	39 (66%)
Polymicrobial infection, *n* (%)	20 (34%)
Methicillin-*susceptible Staphylococcus aureus*, *n* (%)	31 (53%)
Methicillin-*resistant Staphylococcus aureus*, *n* (%)	4 (7%)
*Escherichia coli*, *n* (%)	7 (12%)
*Pseudomonas aeruginosa*, *n* (%)	4 (7%)
*Cutibacterium acnes*, *n* (%)	2 (3%)
**Biologic Characteristics**	
GFR, mL/min/1.73m² (mean ± SD) CLcr (mL/min)	104.8 ± 46.4 120.2 ± 74.35
CRP, mg/L (mean ± SD)	38.5 ± 41.3
Bilirubin, µmol/L (mean ± SD)	9.6 ± 7.5
Proteinemia, g/L (mean ± SD)	64.7 ± 8.6
SGOT, IU/L (mean ± SD)	25.1 ± 24.3
SGPT, IU/L (mean ± SD)	24.4 ± 23.4
Alkaline phosphatase, IU/L (mean ± SD)	135.2 ± 81.8
LEVO, mg/l (mean ± SD)	5.52 ± 4.41

*n*: number of values; SD: standard deviation; BW: body weight; GFR: glomerular filtration rate (calculated by the MDRD formula); CLcr: creatinine clearance (calculated by the Cockcroft–Gault equation); CRP: C-reactive protein; LEVO: observed levofloxacin concentration.

**Table 2:** Mean parameter estimates and relative standard deviations for the final model.

Parameter	Original Dataset		Bootstrap	
	Mean Estimate	RSE (%)	Mean Estimate	RSE (%)
Ka (h^−1^)	0.895	34	0.948	38
CL/F (L/h)	6.10	6	6.00	6.8
V/F (L)	90.6	8	88.4	9.9
Θ_GFR,CL_	0.48	41	0.54	31
Θ_AGE,CL_	−0.52	28	−0.47	42
ω_CL/F_	0.157	19	0.155	22
ω_V/F_	0.061	43	0.043	65
Cov_CL,V_	0.043	NE	0.047	72
σ	0.118	13	0.113	13

RSE: Relative standard error; Ka: constant rate of absorption, CL/F: apparent clearance, V/F: apparent distribution volume, θ_GFR,CL_: influential factor of GFR on CL/F, θAGE_,CL_: influential factor of age on CL/F, ω_CL_: interindividual variability of CLF, ω_V_: interindividual variability of V/F, Cov_CL,V_: covariance between the etas of CL/F and V/F, σ: proportional residual variability.

**Table 3:** Simulated mean ± standard deviation of levofloxacin area under the curve (AUC) (in mg h/L) for a 750 mg per day dose, according to age and glomerular filtration rate.

AGE (year)			GFR (mL/min/1.73 m²)
30	50	70	
147 ± 59	196 ± 82	233 ± 97	**40**
115 ± 45	148 ± 60	176 ± 73	**70**
98 ± 40	128 ± 52	154 ± 65	**100**
84 ± 33	109 ± 43	132 ± 52	**130**
79 ± 35	100 ± 42	122 ± 51	**160**

**Table 4:** Probability (%) to achieve a target AUC/minimum inhibitory concentration (MIC) = 100 for a daily dose of 750 mg and a MIC = 1 mg/L, according to age and glomerular filtration.

AGE (year)			GFR (mL/min/1.73 m²)
30	50	70	
80	93	97	**40**
56	81	91	**70**
40	65	84	**100**
26	52	69	**130**
20	40	62	**160**

**Table 5:** Probability (%) to achieve a target AUC/MIC = 100 for a daily dose of 750 mg and a MIC = 0.5 mg/L, according to age and glomerular filtration.

AGE (year)			GFR (mL/min/1.73 m²)
30	50	70	
99	100	100	**40**
99	99	100	**70**
94	98	99	**100**
87	97	99	**130**
81	95	98	**160**

## Supplementary Materials

The following are available online at https://www.mdpi.com/2079-6382/9/7/401/s1, Table S1: Consistency between the values of levofloxacin clearance obtained in previous population pharmacokinetic (PK) studies and the values calculated with the present model, assuming a bioavailability of 100% and using the mean values of the covariates provided in the corresponding reference (when available).
