# Supplementary material for: Influence of Renal Function and Age on the Pharmacokinetics of Levofloxacin in Patients with Bone and Joint Infections
Source: Antibiotics (Basel). 2020 Jul 10;9(7):401. doi: 10.3390/antibiotics9070401 (PMC7399966; doi:10.3390/antibiotics9070401)
Supplement: Supplementary file 1 [file antibiotics-09-00401-s001.pdf]

Supplemental Table S1: Consistency between the values of levofloxacin clearance obtained in previous population pharmacokinetic (PK) studies and the values calculated with the present model, assuming a bioavailability of 100% and using the mean values of the covariates provided in the corresponding reference (when available).

Clinical context	Mean Age (years)	Mean CLcr[a] or GFR[b] (mL/min or mL/min/1.73 m²)	Mean observed CL/F or CL (L/h)	CL/F or CL* calculated with the present final model (L/h)	CL/F or CL* calculated with the model based on CLcr	Reference
osteo-articular infections (oral route)	57.5	104.8[b]	6.10			Present Study
Severe community acquired infections (IV route)	46.9	86.4[a]	9.27	6.20	5.32	[16]
Critically ill patients (IV route)	61	70[a]	8.66	4.89	4.56	[26]
Healthy volunteers (oral route)	28	114[b]	10.8	9.27	6.51	[25]
Elderly (IV and oral route)	81.2	30.2[b]	2.53–3.04	2.82	2.47	[14]
Pulmonary tuberculosis (oral route)	44	79[a]	7.63	6.14	4.98	[17]
Healthy volunteers (IV route)	55.7	105.4[a]	7.40	6.24	6.15	[20]
Lower respiratory tract infections (oral route)	41.5	100.2[a]	8.97	7.10	5.93	[18]
Haematological malignancies (IV route)	56.7	93.8[a]	5.80	5.85	5.65	[28]
Acute infections (IV route)	54.4	80.6[b]	6.19	5.55	5.06	[19]
Healthy volunteers (IV route)	24.6	125.9[a]	10.2	10.4	7.00	[30]
Prostatectomy (IV route)	NA	NA	7.27	NC	NC	[22]
Healthy volunteers (oral route)	NA	NA	5.97	NC	NC	[23]

IV: intravenous; CLcr: creatinine clearance (expressed in mL/min); GFR: glomerular filtration rate (expressed in mL/min/1.73 m²); [a]CLcr value expressed in mL/min; [b]GFR value expressed in mL/min/1.73m²; CL/F: apparent clearance of levofloxacin; CL: clearance of levofloxacin; CL*: calculated clearance of levofloxacin assuming a bioavailability of 100%; NA: not available; NC: not calculate.
